# Electrical dry needling as an adjunct to exercise, manual therapy and ultrasound for plantar fasciitis: A multi-center randomized clinical trial

**DOI:** 10.1371/journal.pone.0205405

**Published:** 2018-10-31

**Authors:** James Dunning, Raymond Butts, Nathan Henry, Firas Mourad, Amy Brannon, Hector Rodriguez, Ian Young, Jose L Arias-Buría, César Fernández-de-las-Peñas

**Affiliations:** 1 Department of Physical Therapy, Occupational Therapy, Rehabilitation and Physical Medicine, Universidad Rey Juan Carlos, Alcorcón, Spain; 2 American Academy of Manipulative Therapy Fellowship in Orthopaedic Manual Physical Therapy, Montgomery, Alabama, United States of America; 3 Research Physical Therapy Specialists, Columbia, South Carolina, United States of America; 4 Troop Medical Clinic, Eglin Air Force Base, Florida, United States of America; 5 Universita di Roma Tor Vergata, Italy; 6 TOPS Physical Therapy and Orthopaedics, Phoenix, Arizona, United States of America; 7 Manual Physical Therapy Specialists, El Paso, Texas, United States of America; 8 CORA Physical Therapy, Savannah, Georgia, United States of America; Bern University of Applied Science, SWITZERLAND

## Abstract

**Study Design:**

Randomized, single-blinded, multi-center, parallel-group trial.

**Objectives:**

To compare the effects of adding electrical dry needling into a program of manual therapy, exercise and ultrasound on pain, function and related-disability in individuals with plantar fasciitis (PF).

**Background:**

The isolated application of electrical dry needling, manual therapy, exercise, and ultrasound has been found to be effective for PF. However, no previous study has investigated the combined effect of these interventions in this population.

**Methods:**

One hundred and eleven participants (n = 111) with plantar fasciitis were randomized to receive electrical dry needling, manual therapy, exercise and ultrasound (n = 58) or manual therapy, exercise and ultrasound (n = 53). The primary outcome was first-step pain in the morning as measured by the Numeric Pain Rating Scale (NPRS). Secondary outcomes included resting foot pain (NPRS), pain during activity (NPRS), the Lower Extremity Functional Scale (LEFS), the Foot Functional Index (FFI), medication intake, and the Global Rating of Change (GROC). The treatment period was 4 weeks with follow-up assessments at 1 week, 4 weeks, and 3 months after the first treatment session. Both groups received 6 sessions of impairment-based manual therapy directed to the lower limb, self-stretching of the plantar fascia and the Achilles tendon, strengthening exercises for the intrinsic muscles of the foot, and therapeutic ultrasound. In addition, the dry needling group also received 6 sessions of electrical dry needling using a standardized 8-point protocol for 20 minutes. The primary aim was examined with a 2-way mixed-model analysis of covariance (ANCOVA) with treatment group as the between-subjects variable and time as the within-subjects variable after adjusting for baseline outcomes.

**Results:**

The 2X4 ANCOVA revealed that individuals with PF who received electrical dry needling, manual therapy, exercise and ultrasound experienced significantly greater improvements in first-step morning pain (F = 22.021; P<0.001), resting foot pain (F = 23.931; P<0.001), pain during activity (F = 7.629; P = 0.007), LEFS (F = 13.081; P<0.001), FFI Pain Subscale (F = 13.547; P<0.001), FFI Disability Subscale (F = 8.746; P = 0.004), and FFI Total Score (F = 10.65; P<0.001) than those who received manual therapy, exercise and ultrasound at 3 months. No differences in FFI Activity Limitation Subscale (F = 2.687; P = 0.104) were observed. Significantly (*X*^*2*^ = 9.512; P = 0.023) more patients in the electrical dry needling group completely stopped taking medication for their pain compared to the manual therapy, exercise and ultrasound group at 3 months. Based on the cutoff score of ≥+5 on the GROC, significantly (*X*^*2*^ = 45.582; P<0.001) more patients within the electrical dry needling group (n = 45, 78%) achieved a successful outcome compared to the manual therapy, exercise and ultrasound group (n = 11, 21%). Effect sizes ranged from medium to large (0.53<SMD<0.85) at 3 months in favor of the electrical dry needling group.

**Conclusion:**

The inclusion of electrical dry needling into a program of manual therapy, exercise and ultrasound was more effective for improving pain, function and related-disability than the application of manual therapy, exercise and ultrasound alone in individuals with PF at mid-term (3 months).

**Level of evidence:**

Therapy, Level 1b.

## Introduction

Plantar fasciitis (PF) is the most common cause of heel pain and is estimated to affect 10% of the general population during their lifetime.[1] During the years 1995 to 2000, PF accounted for 1 million patient visits per year to medical physicians in the United States.[2] In 2007, the cost of treatment for PF in the United States ranged between $192 and $376 million.[3] There is an ongoing debate regarding the proper nomenclature, whether the disorder should be referred to as plantar fasciitis, plantar fasciosis or plantar heel pain. Imaging and histological findings support the premise that plantar “fasciitis” is actually a degenerative “fasciosis” without inflammation;[4, 5] thus, several studies have used the broader and nonspecific term plantar heel pain.[6–9] Nevertheless, several recent trials,[10–17] literature reviews[18–21] and clinical practice guidelines[22] have reverted back to the “well established phrase”[19] and more common clinical term of plantar fasciitis; therefore, this paper will use the term ‘plantar fasciitis’ (PF).

PF is characterized by intense sharp pain over the medial plantar heel with intial steps in the morning or after inactivity, that increases with prolonged weightbearing activities.[8, 18, 20, 22] The etiology remains unclear; however, PF has been categorized as an enthesopathy, i.e. an attachment dysfunction of ligament or tendon to bone, with the enthesis being the interface between the periosteum and plantar aponeurosis and/or the tendon of the flexor digitorum brevis.[8, 19, 20]

The Cochrane review on PF concluded that high quality evidence of efficacy for any one treatment modality is still lacking.[23] Moreover, treatment options for PF remain controversial and the recomemended method of intervention remains inconsistent.[8, 18, 19, 22, 24] Although a recent clinical trial found short-term improvements in heel pain with the use of full-length silicone insoles as a first line of treatment for PF,[25] there is little evidence to support the use of prefabricated or custom orthoses for long-term improvements in heel pain or disability.[22, 26, 27] A recent systematic review suggested that manual therapy (i.e. joint mobilization/manipulation, soft tissue mobilization, manual stretching, trigger point release) may be effective for PF; however, the optimum dosage (i.e. frequency, intensity, duration) of manual therapy remains unclear.[28] Strength training of the intrinsic foot musculature may be effective for improving pain and function in PF.[29] Although the APTA clinical practice guidelines do not recommend ultrasound therapy for PF,[22] a recent systematic review concluded that “the available higher-quality evidence suggests that patients with persistant PF may benefit from therapeutic ultrasound.”[30]

Additionally, several needling approaches have been investigated for PF. For cases of persistent PF, physicians often recommend corticosteroid injections;[8, 31] however, while corticosteroid injections may be useful for managing PF symptoms at short-term, long-term outcomes appear to be lacking.[23, 32, 33] Moreover, steroid injections have been linked with plantar fat pad atrophy, calcaneal osteomyelitis, plantar fascia weakening and rupture.[34–36] Platelet rich plasma treatment has also been found to be effective for PF;[37] nevertheless, platelet rich plasma injections are considered controversial, expensive and are not normally covered by insurance plans.[38]

Although the 2014 APTA clinical practice guidelines[22] concluded that “the use trigger point dry needling cannot be recommended for individuauls with PF,” a recent meta-analysis of seven trials concluded that trigger point dry needling is effective in patients with PF with a pooled estimate effect size of -15.5 points for pain reduction.[39] Needling therapies[11, 39–41] may be a reasonable non-pharmacologic adjunct therapy for the reduction of pain in individuals with PF who are already receiving manual therapy,[6, 28, 40, 42] exercise,[29, 43] and/or electrophysical agents.[30] Needling therapy refers to the insertion of thin monofilament needles without the use of injectate.[44–48] Dry needling is typically used to treat muscles, ligaments, tendons, subcutaneous fascia, scar tissue, peripheral nerves, and neurovascular bundles for the management of a variety of neuromusculoskeletal pain syndromes.[44, 47–49]

In the absence of high quality evidence for any one treatment modality,[23] it may still be possible to achieve a high success rate with a combination of the treatments for PF.[8, 19] Electrical dry needling,[11, 39–41] manual therapy,[6, 28, 42] exercise[29, 43] and ultrasound,[30, 50] when applied separately, have each been found to be effective for PF. However, no previous study has investigated the combination of the effectiveness of electrical dry needling in addition to manual therapy, exercise and ultrasound in patients with PF. Therefore, the purpose of this multi-center randomized clinical trial was to compare the effects of adding electrical dry needling, into a program of manual therapy, exercise and ultrasound on pain and related-disability in individuals with PF. We hypothesized that individuals receiving electrical dry needling combined with manual therapy, exercise and ultrasound would exhibit greater improvements in pain and related-disability than those receiving only manual therapy, exercise and ultrasound.

## Methods

### Study design

This randomized, single-blinded, multi-center, parallel-group trial compared two treatment protocols for the management of PF: manual therapy, exercise and ultrasound vs. manual therapy, exercise and ultrasound plus electrical dry needling. The primary end-point was first-step pain (when getting out of bed in the morning) as measured by the Numeric Pain Rating Scale (NPRS). Secondary outcomes were resting foot pain intensity (NPRS), pain during activity (NPRS), the Lower Extremity Functional Scale (LEFS), the Foot Functional Index (FFI), medication intake (the number of times the patient had taken prescription or over-the-counter analgesic or anti-inflammtory medication for their PF during the last week), and the Global Rating of Change (GROC).

The current clinical trial was conducted following the Consolidated Standards of Reporting Trials (CONSORT) extension for pragmatic clinical trials. [51] The study was approved by the ethics committee at Universidad Rey Juan Carlos, Madrid, Spain (URJC-DPTO 31–2014) and the trial was prospectively registered (ClinicalTrials.gov: NCT02373618).

### Participants

**Fig 1:**
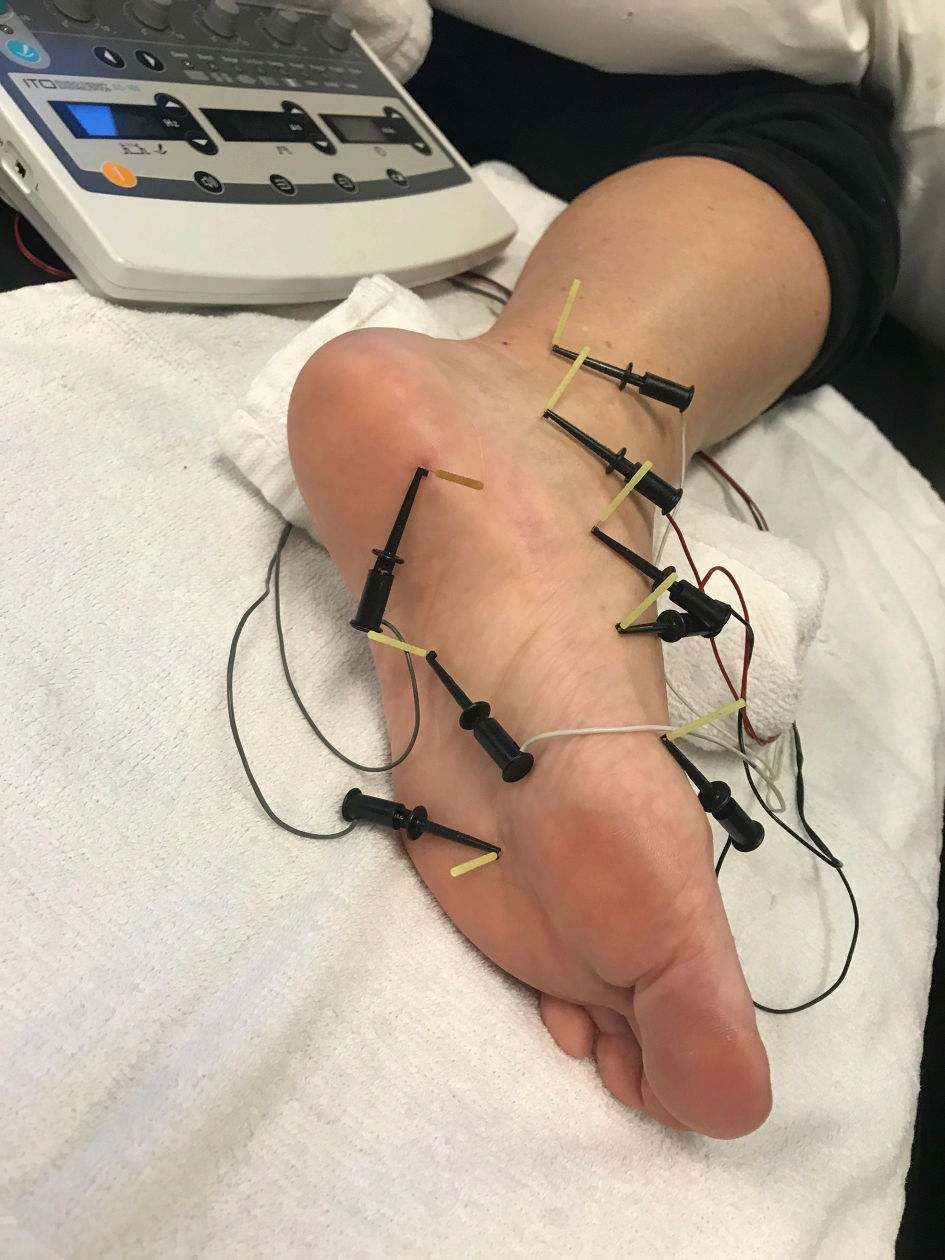
Standardized 8-point protocol of electrical dry needling for PF.

Consecutive individuals with PF from 10 outpatient physical therapy clinics in 6 different states (Arizona, Florida, Georgia, Kentucky, North Carolina, Texas) were screened for eligibility criteria and recruited over a 24-month period (from February 27, 2015 to February 23, 2017). For patients to be eligible, they had to meet the following criteria: 1, a clinical diagnosis of PF in accordance with the clinical practice guidelines from the Orthopaedic Section of the American Physical Therapy Association (APTA);[22] 2, plantar heel pain for longer than 3 months; 3, first-step pain in the morning during the previous week rated at least 2 on the numeric pain rating scale (NPRS 0–10);[52] and 4, aged 18 years or older. Patients were excluded if any of the following criteria were present: 1, a history of surgery to the ankle, foot or lower leg; 2, potential contraindications to manual therapy, dry needling, exercise or ultrasound; 3, had received conservative treatment (i.e. physical therapy, acupuncture, massage therapy, chiropractic treatment or local steroid injections) for PF in the previous 4 weeks; 4, presented with 2 or more positive neurologic signs consistent with nerve root compression; 5, other causes of heel pain (including tarsal tunnel syndrome, calcaneal fracture, ankle or foot instability, arthritis of the foot or ankle, rheumatoid arthritis, spondyloarthropathy, gout, neurogenic claudication, peripheral neuropathy); or 6, had involvement in litigation or worker’s compensation regarding their heel pain. Patients were also excluded if they were pregnant. All participants signed an informed consent prior to their participation in the study. The individual in **Fig 1**has given written informed consent (as outlined in PLOS consent form) to publish a picture of their foot and lower leg.

### Treating therapists

Ten physical therapists (mean age, 34.5 years, SD 5.4) participated in the delivery of treatment for patients in this study. They had an average of 8.0 (SD 4.4) years of clinical experience, and all had completed a 54-hour post-graduate certification program that included practical training in electrical dry needling for PF. In addition, all physical therapists delivering treatment were Fellows-in-Training within the APTA-accredited American Academy of Manipulative Therapy Fellowship in Orthopaedic Manual Physical Therapy post-graduate program, and therefore received advanced clinical training in the diagnosis and treatment of PF. Similar to our previous study[53] and to ensure all examination, outcome assessments, and treatment procedures were standardized, all participating physical therapists were required to study a manual of standard operating procedures and participate in a 6-hour training session with the principal investigator.

### Examination procedure

All patients provided demographic information and completed a number of self-reported measures followed by a standardized history and physical examination at baseline. Participants received a standardized physical examination during which the affected foot, ankle and lower extremity were examined for conditions other than PF; that is, other causes of heel pain were ruled out. The physical examination included, but was not limited to, measurements for the impairments of reduced ankle dorsiflexion range of motion[54] and tightness of the calf and hamstrings.[55] Most of time the diagnosis of PF is straightforward;[19] however, in cases where pain localization is poor, differential diagnosis includes tarsal tunnel syndrome, entrapment of the first branch of the lateral plantar nerve, subtalar arthritis, S_1_ nerve root impingement, fat pad atrophy, proximal plantar fibroma, fat pad contusion, calcaneal bone bruise, or calcaneal stress fractures.[8, 19, 20, 22]

### Randomization and blinding

Following baseline examination, patients were randomly assigned to receive manual therapy, exercise and ultrasound alone or combined with electrical dry needling. Similar to our previous trials,[53, 56, 57] concealed allocation was conducted using a computer-generated randomized table of numbers created by a statistician who was not otherwise involved in the trial and did not participate in analysis or interpretation of the results. Individual and sequentially numbered index cards with the random assignment were prepared for each of the 10 data collection sites. The index cards were folded and placed in sealed opaque envelopes. Blinded to the baseline examination, the treating therapist opened the envelope and proceeded with treatment according to the group assignment. Patients were instructed not to discuss the particular treatment procedure received with the examining clinician. The examining clinician remained blinded to the patient’s treatment group assignment at all times; however, based on the nature of the interventions it was not possible to blind patients or treating therapists.

### Interventions

All participants received up to eight treatment sessions at a frequency of once or twice per week over a 4-week period. Both groups received an impairment-based manual therapy approach directed to the lower limb,[6, 13, 26, 28, 58] an exercise program[7, 12, 13, 29, 42, 43] including self-stretching of the plantar fascia and the Achilles tendon and strengthening exercises for the intrinsic muscles of the foot, and therapeutic ultrasound[6, 30, 50] (Sonicator, Mettler Electronics, Anaheim, CA; 3 MHz, 1.5 W/cm^2^, 20% duty cycle for 5 minutes to the most tender region of the proximal portion of the plantar fascia). Details regarding the manual therapies and exercise program are provided in **S2 Table**.

The exercise program was taught to the patient by an experienced physiotherapist on the first treatment session and supervised on subsequent sessions. Strengthening and stretching exercises were gradually progressed according to tolerance of each individual patient. That is, progression only occurred if the patient reported a decrease in symptoms associated with PF and in the absence of excessive soreness, defined as soreness lasting longer than a few hours post-treatment. Specific details regarding the exercise and manual therapy program are provided in **S2 Table**. Notably, the findings of a recent systematic review suggest that manual therapy (i.e. joint mobilization/manipulation, soft tissue mobilization, manual stretching, trigger point pressure release) may be effective for PF; however, the dose (i.e. frequency, intensity, duration) of manual therapy remains unclear.[28] Further, no clear conclusions can be drawn regarding the most effective stretch position, duration, frequency or optimum number of repetitions; however, plantar fascia stretching may be more effective than Achilles tendon stretching, at least at short term.[42]

All patients in both groups were instructed to complete a home exercise program during the 4-week treatment period. The home exercise program consisted of the same strengthening and stretching exercises that were prescribed and supervised in the clinic, but without supervision. Patients were told to complete the home exercise program 3 times daily on the days that they did not receive supervised physical therapy in the clinic.[6, 26, 41] Patients were asked to monitor their compliance with the home exercise program by maintaining a home exercise program logbook.

In addition to manual therapy, exercise and ultrasound, patients allocated to the dry needling group also received up to 8 sessions of electrical dry needling at a frequency of 1–2 times per week for 4 weeks using a standardized protocol of 8 points for 20 minutes.[39–41, 59, 60]. Within both groups, fewer treatment sessions could be delivered by the treating therapist if symptom resolution occured sooner. Electrical dry needling included an 8-point standardized protocol as depicted in **Fig 1**. Each needle insertion site, angulation and anatomical target is summarized in **S2 Table**. Notably, PF has been categorized as an enthesopathy, with the enthesis being the interface between the periosteum and ligament (plantar aponeurosis) or tendon (flexor digitorum brevis);[8, 19, 20] thus, the primary target for dry needling was the insertion of the plantar fascia at or near the medial tubercle of the calcaneus.[11, 41, 61, 62] Therefore, “periosteal stimulation” or “periosteal pecking” at or near the proximal attachment of the plantar fascia at the medial tubercle of the calcaneus,[63, 64] was performed for 30 seconds over the most painful tender point at the medial calcaneal tubercle.[11, 65, 66] In addition to the 8-point standardized protocol, clinicians were also permitted to insert needles at up to 4 additional locations in the foot and/or lower leg based on the presence of trigger points, or the report of sensitivity by the patient. Notably, the medial head of the gastrocnemius was recommended as one of the four optional needle insertion sites.[59, 61]

Sterilized disposable stainless steel acupuncture needles were used with three sizes: 0.18 mm x 15 mm, 0.25 mm x 30 mm, 0.30 mm x 40 mm. The plantar and medial surface of the foot and ankle were cleaned with alcohol. The depth of needle insertion ranged from 10 mm to 35 mm depending on the point selected (intramuscular, periosteal, perineural) and the patient’s constitution (i.e. size and bone depth, muscle and/or connective tissue thickness). Following insertion, needles were manipulated bi-directionally to elicit a sensation of aching, tingling, deep pressure, heaviness or warmth.[59, 60, 67, 68] The needles were then left in situ for 20 mins[40, 41, 59, 62] with electric stimulation (ES-160 electrostimulator ITO co.) in pairs to all 8 of the obligatory needles using a low frequency (2 Hz), moderate pulse duration (250 microseconds), biphasic continuous waveform at an intensity described by the patient as “mild to moderate”.[40, 41] In cases of bilateral PF, both feet were treated, but only the most painful side at baseline was recorded and analyzed through-out the study to satisfy the assumption of independent data.[9, 69]

### Outcome measures

Among all outcomes included in the clinical trial registry, the primary outcome of the current trial was first-step pain[9, 12, 13, 40, 50, 70] during the morning as measured by the Numeric Pain Rating Scale (NPRS). Patients were asked to indicate the average intensity of first-step pain when getting out of bed in the morning over the past week using an 11-point scale ranging from 0 (“no pain”) to 10 (“worst pain imaginable”) at baseline, 1 week, 4 weeks, and 3 months following the initial treatment session.[71] The NPRS is a reliable and valid instrument to assess pain intensity.[72–74] The MCID for the NPRS has been shown to be 1.74 in patients with a variety of chronic pain conditions;[74] thus, a change of 2 points or a 30% decrease in pain from baseline can be considered as a MCID in individuals with chronic musculoskeletal pain.[74, 75] Furthermore, the MCID in individuals with PF for the VAS (0–100 mm) has been found to be 19 mm for first-step pain and 8 mm for average pain.[76] When compared with the VAS, the NPRS has higher compliance rate, better responsiveness and ease of use, and less practical difficulties;[77, 78] thus, for these reasons we used the NPRS and chose to only include patients with a score of 2 points or greater for first-step pain.[9]

Secondary outcomes included resting mean foot pain (NPRS), pain during activity (NPRS), the Lower Extremity Functional Scale (LEFS), the Foot Functional Index (FFI), medication intake, and the Global Rating of Change (GROC) and were collected at baseline, 1 week, 4 weeks and 3 months after the initial treatment. The Lower Extremity Functional Scale (LEFS) is a commonly used outcome measure in patients with PF[6, 13, 22] and has been found to have excellent validity, test-retest reliability, and responsiveness to change in patients with lower extremity disorders.[79–81] The LEFS consists of 20 questions involving everyday functional activities each worth 0–4 points; therefore, the range for the LEFS is 0–80 points, with higher scores indicating greater levels of function.[80] The MCID for the LEFS has been reported to be 9 points.[80]

The FFI was developed to measure the impact of foot pathology on pain, disability and activity limitation.[82] The FFI is the most widely used foot-specific self-reporting measure[83] and is a commonly used outcome measure in patients with PF.[22, 32, 41] The FFI has been shown to be valid, reliable and sensitive to change in various populations with a variety of foot and ankle disorders.[83, 84] Subscale scores range from 0% to 100%, with higher scores indicating lower levels of function and poorer foot health-related quality of life.[82] The FFI Total Score is derived by calculating the mean of the 3 subscale scores.[82] In patients with PF, the MCID (on a 0 to 100 scale) has been reported to be 12.3%, 6.7% and 6.5% for the Pain Subscale, Disability Subscale, and Total Score, respectively.[85] Notably, the MCID for the FFI Activity Limitation Subscale was reported to be 0.5%, indicating that for a condition like PF, interpretation of this subscale alone is likely inappropriate.[85]

Medication intake was measured as the number of times the patient had taken prescription or over-the-counter analgesic or anti-inflammatory medication in the past week for their heel pain, with five options: (1) not at all, (2) once a week, (3) once every couple of days, (4) once or twice a day, or (5) three or more times a day. Medication intake was assessed at baseline and at 3 months after the first treatment session.

In addition, 1 week, 4 weeks and 3 months following the initial treatment session, patients completed a 15-point GROC question based on a scale described by Jaeschke et al.[86] The scale ranges from -7 (a very great deal worse) to zero (about the same) to +7 (a very great deal better). Intermittent descriptors of worsening or improving are assigned values from -1 to -6 and +1 to +6, respectively. The MCID for the GRC has not been specifically reported but scores of +4 and +5 have typically been indicative of moderate changes in patient status.[86] The GROC may not correlate with changes in function in populations with hip and ankle injuries;[87] nevertheless, it has been used in a number of PF studies.[6, 88, 89]

### Treatment side effects

Patients were asked to report adverse events that they experienced during any part of the study. In the current study, an adverse event was defined as a sequelae of one-week duration with any symptom perceived as distressing and unacceptable to the patient that required further treatment.[90] Particular attention was given to the presence of ecchymosis and post-needling soreness within the group receiving electrical dry needling.

### Sample size determination

The sample size calculations were based on detecting treatment differences of 2 points on the main outcome (MCID for NPRS on first-step pain), assuming a standard deviation of 3 points, a 2-tailed test, an alpha level (α) of 0.05 and a desired power (β) of 90%. The estimated desired sample size was calculated to be at least 49 subjects per group. A dropout percentage of 10% was expected, so 53 patients were included in each group.

### Statistical analysis

Statistical analysis was performed using SPSS software, version 24.0 (Chicago, IL, USA) and it was conducted according to intention-to-treat analysis for patients in the group to which they were first allocated. Mean, standard deviations and/or 95% confidence intervals were calculated for each variable. The Kolmogorov-Smirnov test revealed a normal distribution of the variables (P>0.05). Baseline demographic and clinical variables were compared between both groups using independent Student t-tests for continuous data and χ2 tests of independence for categorical data.

The effects of treatment on first-step pain intensity, resting foot pain, pain during activity, physical function, and related-disability were each examined with a 2-by-4 mixed model analyses of covariance (ANCOVA) with treatment group (manual therapy, exercise and ultrasound versus manual therapy, exercise and ultrasound plus electrical dry needling) as the between-subjects factor, time (baseline, 1 week, 4 weeks and 3 months follow-up) as the within-subjects factor, and adjusted for baseline data for evaluating between-groups differences. Separate ANCOVAs were performed with first-step pain intensity (NPRS), mean heel pain at rest (NPRS), pain during activity (NPRS), the Lower Extremity Functional Scale (LEFS), the FFI Total Score, the FFI Pain Subscale, the FFI Disability Subscale, and the FFI Activity Limitation Subscale as the dependent variable. For each ANCOVA, the main hypothesis of interest was the 2-way interaction (group by time) with a Bonferroni-corrected alpha of 0.0125 (4 time points). We used χ2 tests to compare self-perceived improvement with GROC and changes in medication intake. To enable comparison of between-group effect sizes, standardized mean score differences (SMDs) were calculated by dividing mean score differences between groups by the pooled standard deviation. Numbers needed to treat (NNT) and 95% confidence intervals (CI) were also calculated at the 3-months follow-up period using each definition for a successful outcome.

## Results

**Fig 2:**
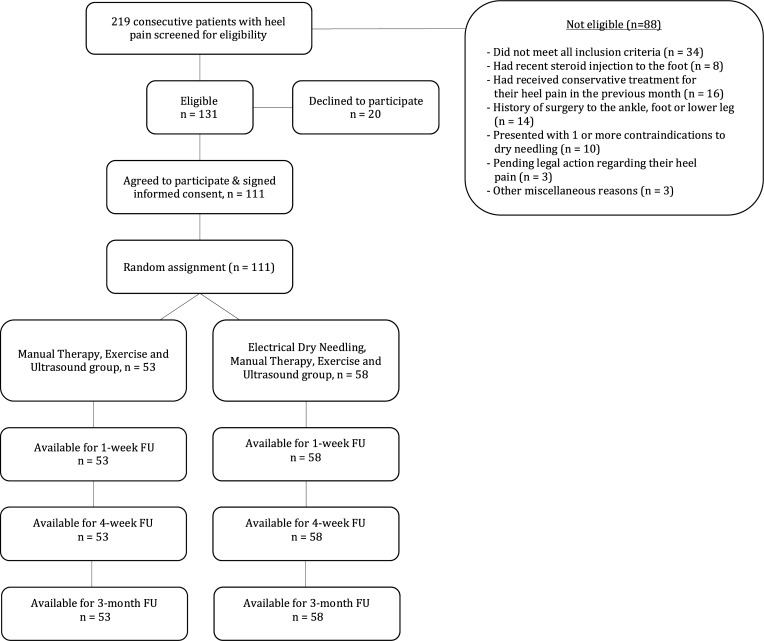
Flow diagram of patient recruitment and retention.

Between February 2015 and February 2017, 219 consecutive patients with PF were screened for eligibility criteria. One hundred eleven (50.7%) satisfied all the inclusion criteria, agreed to participate, and were randomly allocated into the manual therapy, exercise and ultrasound (n = 53) group or the manual therapy, exercise, ultrasound plus electrical dry needling (n = 58) group. Randomization resulted in similar baseline characteristics for all variables (**Table 1**). The reasons for ineligibility are found in **Fig 2**, which provides a flow diagram of patient recruitment and retention. No patients were lost at any of the follow-up periods in either group. None of the participants in any group reported receiving other interventions during the study, excluding the use of NSAIDs, as needed and recorded. There was no significant difference (P = 0.432) between the mean number of completed treatment sessions for the manual therapy, exercise and ultrasound group (mean: 6.2) and the manual therapy, exercise and ultrasound plus electrical dry needling group (mean: 5.9). One hundred three patients (92.8%) reported compliance with the home exercise program.

**Table 1:** Baseline characteristics by treatment assignment.

Baseline Variable	Manual Therapy + Exercise + Ultrasound (n = 53)	Manual Therapy + Exercise + Ultrasound + Electrical Dry Needling (n = 58)
**Gender (male/female)**	27 / 26	37 / 21
**Age (years)**	42.6 ± 11.6	39.1 ± 10.4
**Weight (kg)**	81.6 ± 16.3	81.9 ± 14.3
**Height (cm)**	172.0 ± 9.1	173.4 ± 9.2
**Duration of symptoms (days)**	336.4 ± 288.8	386.1 ± 451.1
**Medication intake n (%)**		
**Not at all**	28 (52.8%)	26 (44.8%)
**Once a week**	2 (3.8%)	5 (8.6%)
**Once every couple of days**	15 (28.3%)	18 (31.0%)
**Once or twice a day**	8 (15.1%)	7 (12.1%)
**Three or more times a day**	0 (0%)	2 (3.4%)
**Number of treatment sessions**	6.2 ± 2.4	5.9 ± 2.5
**Mean intensity of heel pain (NPRS, 0–10)**	6.1 ± 1.6	5.8 ± 1.8
**First step pain intensity (NPRS, 0–10)**	6.4 ± 1.8	6.3 ± 2.0
**Pain intensity during activity (NPRS, 0–10)**	5.8 ± 2.1	5.5 ± 2.1
**LEFS (0–80)**	48.9 ± 13.1	50.4 ± 12.8
**FFI Pain Scale (0–100)**	59.3 ± 16.2	57.8 ± 19.8
**FFI Disability Scale (0–100)**	50.7 ± 18.4	44.9 ± 24.3
**FFI Activity Limitation Scale (0–100)**	16.3 ± 12.2	15.1 ± 12.6
**FFI Total Score (0–100)**	42.1 ± 12.7	39.3 ± 16.9

Data are mean (SD) except for gender and medication intake. NPRS = Numeric Pain Rating Scale, 0–10, lower scores indicate less pain; LEFS = Lower Extremity Functional Scale, 0–80, higher scores indicating greater levels of function; FFI = Foot Functional Index, 0–100%, higher scores indicating lower levels of function and poorer foot health-related quality of life.

Thirty-nine patients assigned to the manual therapy, exercise and ultrasound plus electrical dry needling group (67.2%) experienced post-needling muscle soreness and 15 (25.9%) experienced mild bruising (ecchymosis) which most commonly resolved spontaneously within 48 hours and 2–4 days, respectively. In addition, one patient (1.7%) in the electrical dry needling group experienced drowsiness, headache or nausea, which spontaneously resolved within several hours. No other adverse events were reported.

**Fig 3:**
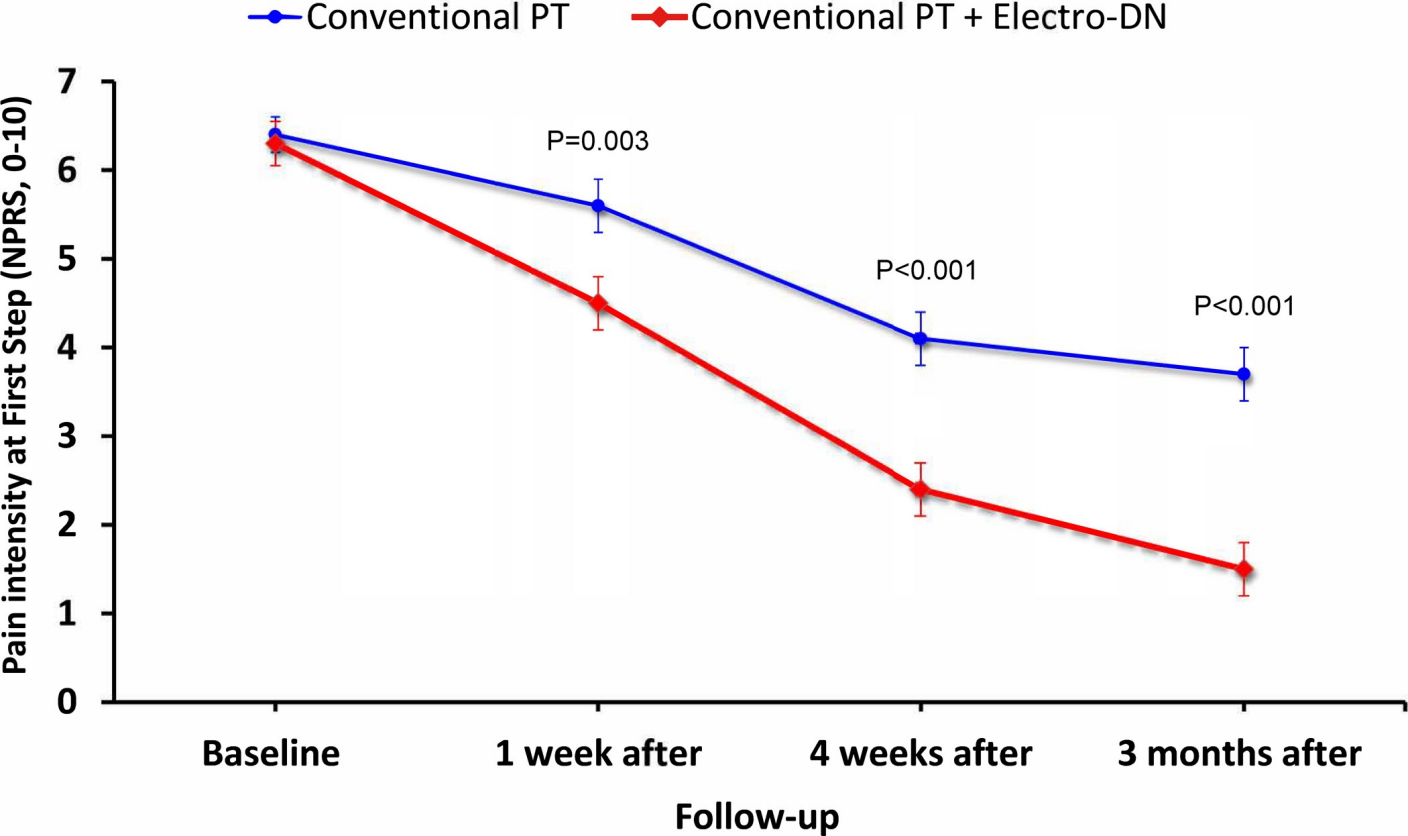
Evolution of first step pain intensity (NPRS) throughout the course of the study stratified by randomized treatment assignment. Data are means (standard error).

Adjusting for baseline outcomes, the mixed-model ANCOVA revealed a significant Group*Time interaction for the primary outcome (F = 22.021; P<0.001): patients receiving electrical dry needling experienced significantly greater improvements in first-step morning pain at 4 weeks (Δ -1.6, 95%CI -2.4 to -0.8, P<0.001) and 3 months (Δ -2.2, 95%CI -2.8 to -1.6, P<0.001) than those receiving manual therapy, exercise and ultrasound alone (**Fig 3**). Between-groups effect sizes were medium (SMD: 0.68) at 4 weeks and large (SMD: 0.85) at 3 months after the first treatment session in favor of the dry needling group.

**Fig 4:**
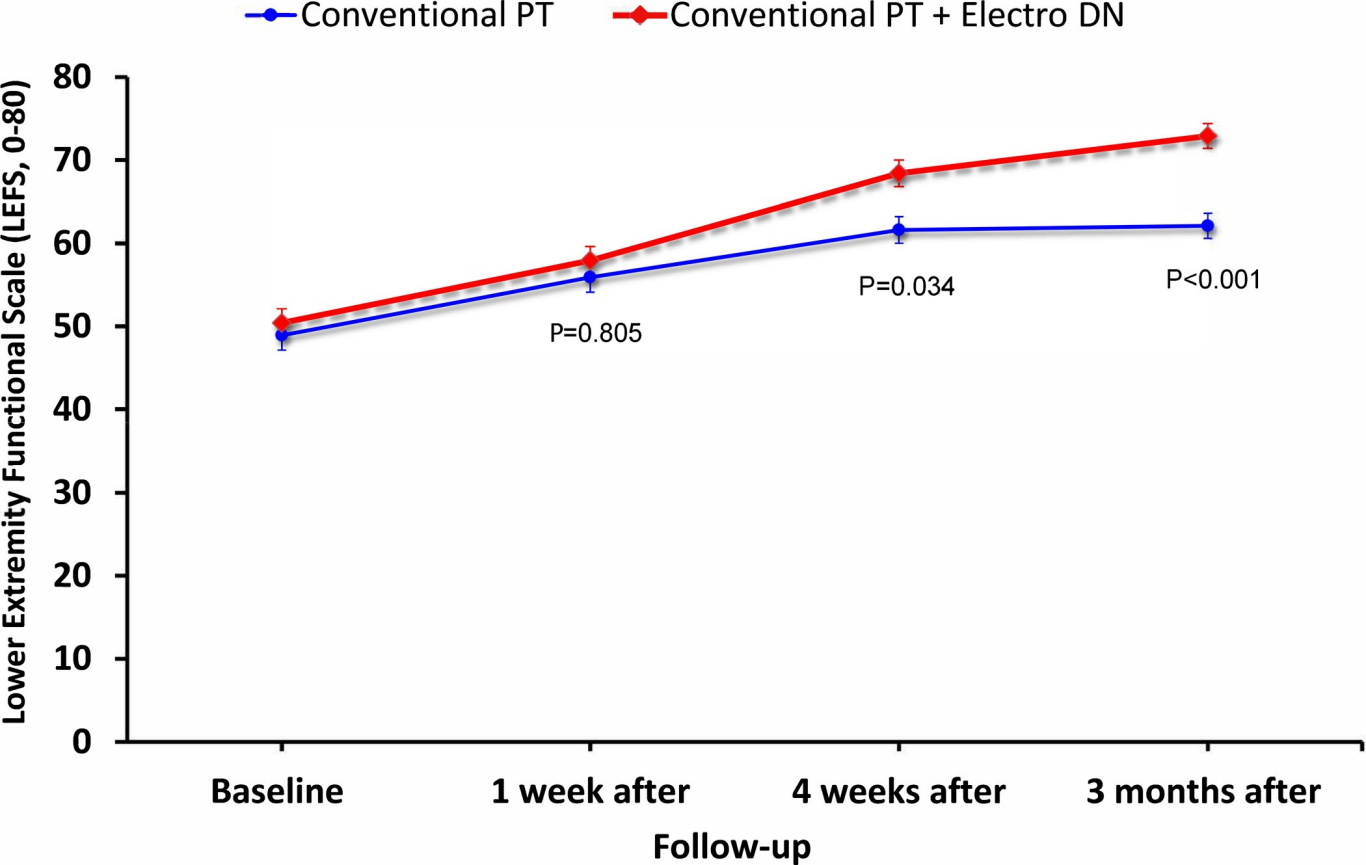
Evolution of lower extremity function (LEFS, 0–80) throughout the course of the study stratified by randomized treatment assignment. Data are means (standard error).

Similarly, significant Group*Time interactions were also found for resting foot pain (F = 23.931; P<0.001), pain during activity (F = 7.629; P = 0.007), LEFS (F = 13.081; P<0.001; **Fig 4**), FFI Pain Subscale (F = 13.547; P<0.001), FFI Disability Subscale (F = 8.746; P = 0.004), and FFI Total Score (F = 10.676; P<0.001), but not for FFI Activity Limitation Subscale (F = 2.687; P = 0.104), in favor of dry needling (**Table 2**). For the LEFS, FFI Total and all significant FFI Subscales, between-groups effect sizes were small to medium (0.32<SMD<0.55) at 4 weeks and medium (0.53<SMD<0.66) at 3 months after the first treatment session in favor of the dry needling group (**Table 3**). A 3-way mixed model ANCOVA (i.e. Group*Time*Treating Therapist) was used to determine if the between-subjects variable of treating therapist had any effect on the results. That is, there was no significant effect of different treating therapists on first step pain (F = 1.447; P = 0.232), resting foot pain (F = 2.391, P = 0.125) or disability (LEF: F = 1.195, P = 0.277; FFI Total: F = 2.168, P = 0.144).

**Table 2:** Foot Functional Index (FFI) within-group and between-groups mean scores by randomized treatment assignment.

Outcomes	Timeline Scores: Mean ± SD (95% CI) Within-Group Change Scores: Mean (95% CI)		Between-Group Differences: Mean (95% CI)
	MT + EX + US (n = 53)	MT + EX + US + EDN (n = 58)	
**Foot Functional Index—Pain Scale (0–100)**			
**Baseline**	59.3 ± 16.2 (54.3, 64.3)	57.8 ± 19.8 (53.0, 62.6)	
**1 week**	51.5 ± 17.9 (45.9, 57.1)	48.7 ± 22.9 (43.3, 54.1)	
Change baseline → 1 week	-7.8 ± 15.0 (-11.9, -3.7)	-9.1 ± 14.6 (-12.9, 5.3)	-1.3 (-6.9, 4.3); P = 0.665
**4 weeks**	40.3 ± 21.6 (34.9, 45.7)	27.4 ± 18.6 (22.1, 32.7)	
Change baseline → 4 weeks	-19.0 ± 20.4 (-24.6, -13.4)	-30.4 ± 19.3 (-35.5, -25.3)	-11.4 (-18.8, -4.0); P = 0.003
**3 months**	34.6 ± 22.2 (28.9, 40.3)	19.2 ± 20.1 (13.7, 24.7)	
Change baseline → 3 months	-24.7 ± 21.0 (-30.5, 18.9)	-38.6 ± 21.4 (-44.2, -33.0)	-13.9 (-21.8, -6.0); P = 0.001
**Foot Functional Index—Disability Scale (0–100)**			
**Baseline**	50.7 ± 18.4 (44.8, 56.6)	44.9 ± 24.3 (39.3, 50.5)	
**1 week**	41.3 ± 18.9 (35.2, 47.4)	37.6 ± 25.2 (31.8, 43.4)	
Change baseline → 1 week	-9.4 ± 16.3 (-13.9, -4.9)	-7.3 ± 14.7 (-11.2, -3.4)	2.2 (-3.6, 9.0); P = 0.460
**4 weeks**	30.9 ± 20.2 (25.9, 35.9)	18.4 ± 16.5 (13.6, 23.2)	
Change baseline → 4 weeks	-19.8 ± 19.0 (-25.0, -14.6)	-26.5 ± 22.0 (-32.3, -20.7)	-6.7 (-14.4, 1.0); P = 0.092
**3 months**	29.3 ± 21.0 (24.3, 34.3)	11.5 ± 15.6 (6.7, 16.3)	
Change baseline → 3 months	-21.4 ± 20.8 (-27.2, -15.6)	-33.4 ± 23.2 (-39.5, -27.3)	-12.0 (-20.3, -3.7); P = 0.005
**Foot Functional Index—Activity Limitation Scale (0–100)**			
**Baseline**	16.3 ± 12.2 (12.9, 19.7)	15.1 ± 12.6 (11.9, 18.3)	
**1 week**	13.3 ± 11.4 (10.2, 16.4)	12.9 ± 11.2 (9.9, 15.9)	
Change baseline → 1 week	-3.0 ± 7.0 (-4.9, -1.1)	-2.2 ± 7.5 (-4.2, -0.2)	0.8 (-1.9, 3.5); P = 0.547
**4 weeks**	8.1 ± 7.6 (6.0, 10.2)	5.5 ± 7.9 (3.5, 7.5)	
Change baseline → 4 weeks	-8.2 ± 9.9 (-10.9, -5.5)	-9.6 ± 11.4 (-12.6, -6.6)	-1.4 (-5.5, 2.7); P = 0.486
**3 months**	8.5 ± 11.8 (6.1, 10.9)	3.7 ± 5.4 (1.3, 6.1)	
Change baseline → 3 months	-7.8 ± 12.8 (-11.3, -4.3)	-11.4 ± 11.9 (-14.6, -8.2)	-3.6 (-8.3, 1.1); P = 0.118
**Foot Functional Index–Total Score (0–100)**			
**Baseline**	42.1 ± 12.7 (38.0, 46.2)	39.3 ± 16.9 (35.3, 43.3)	
**1 week**	35.4 ± 13.5 (30.9, 39.9)	33.1 ± 18.6 (28.8, 37.4)	
Change baseline → 1 week	-6.7 ± 10.7 (-9.7, 3.7)	-6.2 ± 9.9 (-8.8, -3.6)	0.5 (-4.4, 3.4); P = 0.761
**4 weeks**	26.5 ± 14.6 (22.7, 30.3)	17.2 ± 12.8 (13.5, 20.9)	
Change baseline → 4 weeks	-15.6 ± 14.2 (-19.6, -11.6)	-22.1 ± 15.4 (-26.2, -18.0)	-6.5 (-12.1, -0.9); P = 0.023
**3 months**	24.2 ± 16.3 (20.2, 28.2)	11.5 ± 12.9 (7.6, 15.4)	
Change baseline → 3 months	-17.9 ± 16.0 (-22.4, -13.4)	-27.8 ± 16.8 (-32.2, -23.4)	-9.9 (-16.0, -3.8); P = 0.002

**Table 3:** Between-group effect sizes (SMD) in favor of the dry needling group when compared to conventional physical therapy (manual therapy, exercise and ultrasound).

Outcome	1^st^ Step Pain	LEFS	FFI Total	FFI Pain	FFI Disability	FFI Activity Limitation
4 weeks	0.68	0.40	0.43	0.55	0.32	0.13
3 months	0.85	0.66	0.58	0.62	0.53	0.30

Large between-group effect size: Cohen’s *d* = .8 or greater. Medium effect size: Cohen’s *d* = .5 or greater. Small effect size: Cohen’s *d* = .2 or greater. Effect size provides information about the magnitude or strength of the difference between the two groups.

Significantly (*X*^*2*^ = 9.512; P = 0.023) more patients in the electrical dry needling group (n = 47, 81%) completely stopped taking medication for their pain compared to the manual therapy, exercise and ultrasound group (n = 37, 69%) at 3 months. Based on the cutoff score of +5 or higher on the GROC, significantly (*X*^*2*^ = 14.887; P<0.001) more patients in the electrical dry needling group (n = 45, 77%) achieved a successful outcome compared to manual therapy, exercise and ultrasound group (n = 11, 21%) at 3 months (**Table 4**). Therefore, based on the cutoff score of ≥+5 on the GROC at 3-month follow-up, the NNT was 1.76 (95%CI: 1.39, 2.41) in favor of the electrical dry needling group. Likewise, based on a 50% improvement from baseline to 3 months in first step morning pain on the NPRS, the NNT was 2.44 (95%CI: 1.75, 4.02) in favor of the electrical dry needling group.

**Table 4:** Self-perceived improvement with Global Rating of Change (GROC) in both groups [n (%)].

Global Rating of Change (GROC, -7 to +7)	Manual Therapy + Exercise + Ultrasound (n = 53)	Manual Therapy + Exercise + Ultrasound + Electrical Dry Needling (n = 58)
**1 week after first treatment session**		
**Moderate changes (+4 / +5)**	8 (15%) / 3 (5.5%)	5 (8.5%) / 4 (7%)
**Large changes (+6 / +7)**	1 (2.0%) / 0 (0%)	3 (5.5%) / 1 (1.5%)
**4 weeks after first treatment session**		
**Moderate changes (+4 / +5)**	7 (13.5%) / 5 (9.5%)	9 (15.5%) / 15 (26%)
**Large changes (+6 / +7)**	3 (5.5%) / 1 (2.0%)	15 (26%) / 5 (8.5%)
**3 months after first treatment session**		
**Moderate changes (+4 / +5)**	8 (15%) / 4 (7%)	6 (10.5%) / 13 (22.5%)
**Large changes (+6 / +7)**	6 (11.5%) / 1 (2.0%)	19 (32.5) / 13 (22.5%)

## Discusssion

### Findings

To our knowledge, this study is the first randomized clinical trial investigating the effectiveness of electrical dry needling combined with manual therapy, exercise and ultrasound in patients with PF. The results suggest that a mean of 6 sessions of manual therapy, exercise, and ultrasound plus electrical dry needling, using an 8-point standardized protocol targeting the foot locally at a frequency of 1–2 times per week over 4 weeks, resulted in greater improvements in pain intensity, function, medication intake, related-disability and foot health-related quality of life than manual therapy, exercise and ultrasound alone. For the primary outcome of first-step morning pain, between-groups effect sizes were medium at 4 weeks and large at 3 months in favor of the dry needling group. The between-groups difference for changes in first step pain at 3 months, as measured by the NPRS (2.2 points, 1.6, 2.8) exceeded the reported MCID.[74]^,^[76] In addition, for function (LEFS), pain (FFI Pain Subscale), disability (FFI Disability Subscale) and foot health-related quality of life (FFI Total), the point estimates for the between-groups difference at 3 months [LEFS (9.26 points); FFI Pain (13.9%); FFI Disability (12.0%); FFI Total (9.9%)] also exceeded the respective MCID (i.e., 9 points[80] for the LEFS; 12.3%[85] for FFI Pain; 6.7%[85] for FFI Disability; 6.5%[85] for FFI Total) for each outcome. Finally, the NNT suggests for every 2 patients treated with electrical dry needling, rather than manual therapy, exercise and ultrasound alone, one additional patient with PF achieves clinically important reductions in first-step pain and related-disability at 3 months.

Similar to the findings of the current study, another randomized controlled trial of patients with chronic PF reported a 69% reduction in foot pain and an 80% success rate following 10 sessions of electroacupuncture over 5 weeks targeting the most tender points over the medial plantar aspect of the calcaneus with 2 to 6 needles left in place for 30 minutes.[41] Likewise, a more recent randomized controlled trial of 84 patients with PF reported statistically significant differences in first-step pain and foot pain in favor of intra-muscular trigger point dry needling over sham dry needling;[9] nevertheless, the between-groups differences in first-step pain and the pain subscale of the Foot Health Status Questionnaire reported in this study did not exceed the MCID.[9]

The underlying mechanisms as to why the electrical dry needling group in the current study experienced greater improvements than the manual therapy, exercise and ultrasound alone group remains to be elucidated. However, mechanical and electric periosteal stimulation, peri-neural needling of the tibial and lateral plantar nerves, and the duration that the needles are left in situ may be important components to consider when using dry needling therapies in patients with PF.

### Rationale for periosteal pecking at the medial tubercle of the calcaneus

Although the etiology of PF remains unclear, the proximal attachment of the plantar aponeurosis at the medial tubercle of the calcaneus is most often reported by patients as the origin of symptoms and the site of greatest discomfort.[91–93] In fact, 3 of the 5 previous studies on dry needling in patients with PF have specifically targeted the insertion of the plantar fascia at or near the medial tubercle of the calcaneus.[41, 61, 62] Therefore, for 1 of the 8 mandatory needle placements, we used periosteal “pecking” or “peppering” to target the enthesis, i.e. the interface between the periosteum and plantar aponeurosis and/or the tendons of the flexor digitorum brevis and quadratus plantae,[8, 19, 20] by tapping the needle repeatedly onto or near the periosteum of the medial tubercle of the calcaneus as this technique has been previously used and appears to be an important component of the needling treatment in patients with PF.[63–66]

Periosteal “pecking” or “peppering”, via mutliple penetrations with dry needles at or near the proximal attachment of the plantar fascia over the medial tubercle of the calcaneus, is intended to stimulate microtrauma and local inflammation,[94] augment the fibroblastic reparative process,[95] increase the concentration and reorganization of collagen fibers,[95–99] and mediate the proliferative and remodeling phase of healing at the interface between the periosteum and plantar aponeurosis (i.e. the enthesis or teno-osseus junction).[99–101] Periosteal stiumulation (i.e. pecking/peppering) has previously been performed in conjunction with injection therapies for PF.[63, 64] In the case of corticosteroid injections, peppering resulted in significantly greater reductions in pain secondary to PF than corticosteroid injection alone.[65, 66] Similarly, another trial found miniscalpel-needle release (“over the most painful tender point at the medial calcaneal tubercle”) was superior to steroid injections at short and long-terms for improving first step morning pain in patients with chronic recalcitrant PF.[11] Additionally, for the management of osteoarthritis of the knee and/or hip, a number of studies have demonstrated that periosteal needling leads to greater improvements in pain and disability than superficial needling approaches that target muscle tissue alone.[102–104]

### Rationale for peri-neural needling

A number of peri-neural needle points were used with electric stimulation to target the posterior tibial nerve, medial plantar nerve, and lateral plantar nerve, which provide the sensory innervation to the plantar surface of the foot and the medial tubercle of the calcaneus.[105] A recent study found ultrasound-guided pulsed-radio frequency energy of the posterior tibial nerve to be a useful strategy for improving the pain and tissue thickness secondary to PF.[106] In a separate study, Arslan et al also reported pulsed-radio frequency ablation of the lateral plantar nerve is an effective technique for reducing pain associated with PF.[107]

### Duration of needle placement

Aside from one cohort study that treated patients with PF with a single lidocaine injection in which the needle was immediately removed upon dispensing the injectate,[61] other studies have left the needles in place for 5 minutes,[9] 15 minutes,[59] 20 minutes,[40, 62] and 30 minutes.[17] Notably, Cotchett et al[9] left the needles in place for a much shorter duration than the others (15–30 minutes[17, 40, 59, 62]) and did not target the insertion of the plantar fascia at or near the medial tubercle of the calcaneus;[41, 61, 62] hence, this may be one reason why the between-groups difference in first-step pain intensity in our study exceeded the MCID, whereas the between-groups difference in first-step pain reported by Cotchett et al[9] did not exceed the MCID for that measure.

### Electrical dry needling vs. manual dry needling

Electrical dry needling (i.e. electroacupunture) has been found to cause the release of substance-P and CGRG predominantly from non-neural structures, facilitating a negative feedback loop to neural and neuroactive components of the target tissue.[108, 109] In the case of periosteal needling, this may lead to decreased inflammation of the densely innervated periosteum—i.e. at the proximal attachment of the plantar aponeurosis at the medial tubercle of the calcaneus which is most frequently reported by patients as the origin of symptoms and the site of greatest discomfort. Notably, CGRP in high quantities causes inflammation, but the concurrent release of substance-P combined with electric stimulation in the vicinity of the periosteum may provide sustained, low levels of CGRP required for a potent anti-inflammatory and therefore anti-nociceptive effect.[110–113] CGRP also initiates a cascade of events mediated by protein kinase A (PKA) in vascular smooth muscle, leading to vasodilation.[114] Moreover, PKA stimulates nitric oxide synthase, increasing the production of nitric oxide, thereby exaggerating the vasodilation effect.[114] The improved vasodilation may improve the microcirculation within the plantar foot, resulting in increased opioid delivery and decreased inflammatory factors in the vicinity of the plantar aponeurosis.[115, 116] Mechanical and electric needle stimulation close to the periosteum of bone may be particularly advantageous, as acupuncture has been shown to reduce IL-6 mRNA expression in bone marrow, thereby limiting inflammation, and inhibiting myelogenic osteoclast activity driving degeneration.[117] Additionally, and although for a different chronic musculoskeletal condition, a recent meta-analysis[118] and a separate secondary analsyis that pooled data from the Cochrane review[119, 120] concluded that electroacupuncture is superior to manual acupuncture in knee osteoarthritis.

### Strengths and limitations

Major strengths of the current study include the inclusion of a large sample size with 10 treating physical therapists from 10 clinics in 6 different geographical states, and the use of the same standardized 8-point needling protocol and dosage paramenters. However, we only assessed mid-term follow-up; thus, we do not know if the significant between-groups differences observed at 3 months would be sustained in the long-term. We also cannot be certain that the results are generalizable to other dry needling protocols, dosages, techniques or needle placements. Additionally, we did not include a dry needling placebo group; which should be included in future studies. Lastly, therapist and patient treatment preferences were not collected and could potentially affect the results.

## Conclusions

The results of the current randomized clinical trial demonstrated that patients with PF who received manual therapy, exercise and ultrasound plus electrical dry needling experienced significantly greater improvements in first-step morning pain intensity, resting heel pain, pain during activity, function, related-disability and foot health-related quality of life, and medication intake as compared to the group that received manual therapy, exercise and ultrasound alone. Future studies should examine the effectiveness of different types and dosages of electrical dry needling and include a long-term follow-up.

## Supporting information

S1 Table CONSORT checklist. (PDF)

S2 Table Appendix 1: Description of electrical dry needling, manual therapy and exercise interventions. (PDF)

S3 Table Plantar fasciitis trial study protocol. (PDF)
