# Supplementary material for: Electrical dry needling as an adjunct to exercise, manual therapy and ultrasound for plantar fasciitis: A multi-center randomized clinical trial
Source: PLoS One. 2018 Oct 31;13(10):e0205405. doi: 10.1371/journal.pone.0205405 (PMC6209187; doi:10.1371/journal.pone.0205405)
Supplement: S2 Table — (PDF) [file pone.0205405.s002.pdf]

S2 Table: Appendix 1: Description of Electrical Dry Needling, Manual Therapy and Exercise Interventions

Technique: 8-point electrical dry needling protocol for plantar fasciitis (PF)

Technique Description: The technique is performed with the patient in prone or side lying. Sterilised disposable stainless steel Seirin J-type acupuncture needles were used with three sizes: 0.18 mm x 15 mm, 0.25 mm x 30 mm, 0.30 mm x 40 mm. The plantar and medial surface of the foot and ankle were cleaned with alcohol. The following 8 needles were inserted **(Fig 2)**:

(1) superoposterior and slightly lateral insertion angle toward the proximal attachment of the plantar fascia at the medial tubercle of the calcaneus. PF has been categorized as an enthesopathy, with the enthesis being the interface between the periosteum and ligament (plantar aponeurosis) or tendon (flexor digitorum brevis); thus, the primary target for dry needling was the insertion of the plantar fascia at or near the medial tubercle of the calcaneus. Therefore, "periosteal stimulation" or "periosteal pecking" at or near the proximal attachment of the plantar fascia at the medial tubercle of the calcaneus, was performed for 30 seconds over the most painful tender point at the medial calcaneal tubercle;

(2) medial to lateral perpendicular insertion within the distal abductor hallucis, immediately plantar and proximal to the head of the first metatarsal—common myofascial trigger point location and perineural for the medial plantar nerve;

(3) medial to lateral perpendicular insertion within the abductor hallucis, immediately plantar and distal to the base of the first metatarsal—common myofascial trigger point location and perineural for the medial plantar nerve;

(4) medial to lateral perpendicular insertion, immediately inferior to the sustentaculum tali (approximately two fingerbreadths inferior to the inferior apex of the medial malleolus)—near the bifurcation point of the tibial nerve and posterior tibial artery;
(5) medial to lateral perpendicular insertion in the depression midway between the prominence of the medial malleolus and the Achilles tendon—perineural point for the tibial nerve at the ankle;
(6) plantar to dorsal perpendicular insertion in the mid belly of flexor digitorum brevis and quadratus plantae muscles, two to three fingerbreadths distal to the anterior and plantar border of the calcaneus—common myofascial trigger point within the flexor digitorum brevis and perineural stimulation for the lateral plantar nerve;
(7) plantar to dorsal perpendicular insertion within the distal plantar aponeurosis near its attachment at the metatarsophalangeal plates, within the depression on sole of the foot one third of the distance from the tip of the second toe to the posterior calcaneus;
(8) medial to lateral perpendicular insertion within the abductor hallucis, within the depression immediately plantar to the navicular tuberosity—common myofascial trigger point and perineural for the medial plantar nerve.

Technique: self-stretching of the plantar fascia

Technique Description: both groups received self-stretching of the plantar fascia in the sitting position, with the patient crossing the affected foot over the contralateral thigh and performed passive extension of the metatarsophalangeal joints.

Technique: self-stretching of the gastrocnemius, soleus and Achilles tendon

Technique Description: both groups received self-stretching of the gastrocnemius, soleus and Achilles tendon. The patient leaned forward in the standing position with the

affected foot farther away from the wall, while keeping the heel on the floor—the soleus muscle was emphasized with the knee flexed and the gastrocnemius muscle with the knee extended.

Technique: nonthrust joint mobilization/manipulation to the lower limb

Technique Description: both groups received an impairment-based manual therapy approach directed primarily to the foot/ankle, but also to the hip and knee, including but not limited to passive anterior to posterior talocrural joint mobilizations in weight-bearing and non-weight-bearing positions and/or distraction thrust talocrural joint manipulation to improve ankle dorsiflexion, subtalar joint lateral glide mobilizations for eversion and inversion, and anterior and posterior first tarsometatarsal joint glide mobilizations for pronation and supination of the midfoot.

Technique: strengthening exercises for the intrinsic muscles of the foot

Technique Description: both groups received strengthening exercises for the intrinsic muscles of the foot including tool curls, toe taps and toe marble/coin pick-ups.
