# Supplementary material for: Electrical dry needling as an adjunct to exercise, manual therapy and ultrasound for plantar fasciitis: A multi-center randomized clinical trial
Source: PLoS One. 2018 Oct 31;13(10):e0205405. doi: 10.1371/journal.pone.0205405 (PMC6209187; doi:10.1371/journal.pone.0205405)
Supplement: S3 Table — (PDF) [file pone.0205405.s003.pdf]

PLANTAR FASCIITIS STUDY PROTOCOL

Background and Purpose

Plantar fasciitis (PF) is the most common cause of heel pain and is estimated to affect 10% of the general population during their lifetime. During the years 1995 to 2000, PF accounted for 1 million patient visits per year to medical physicians in the United States. In 2007, the cost of treatment for PF in the United States ranged between $192 and $376 million. Electrical dry needling, manual therapy exercise and ultrasound, when applied separately, have each been found to be effective for PF. However, no previous study has investigated the combination of the effectiveness of electrical dry needling in addition to manual therapy, exercise and ultrasound in patients with PF. Therefore, the purpose of this multi-center randomized clinical trial is to compare the effects of adding electrical dry needling, into a program of manual therapy, exercise and ultrasound on pain and disability in individuals with PF. We hypothesize that individuals receiving electrical dry needling combined with manual therapy, exercise and ultrasound will exhibit greater improvements in pain and disability than those receiving only manual therapy, exercise and ultrasound.

Study Design

Consecutive individuals with PF from outpatient physical therapy clinics in 6 U.S. states will be screened for eligibility criteria and recruited. For patients to be eligible, they need to meet the following criteria: 1, a clinical diagnosis of PF in accordance with the clinical practice guidelines from the American Physical Therapy Association; 2, plantar heel pain for longer than 3 months; 3, first-step pain in the morning during the previous week rated at least 2 on NPRS; and 4, aged 18 years or older. Patients will be excluded if any of the following criteria are present: 1, a history of surgery to the ankle, foot or lower leg; 2, contraindications to manual therapy, dry needling, exercise or ultrasound; 3, have had physical therapy, acupuncture, massage therapy, chiropractic treatment or local steroid injections for PF in the previous 4 weeks; 4, present with 2 or more positive neurologic signs consistent with nerve root compression; 5, have another cause of heel pain (including tarsal tunnel syndrome, calcaneal fracture, ankle or foot instability, arthritis of the foot or ankle, rheumatoid arthritis, neurogenic claudication, peripheral neuropathy); or 6, have involvement in litigation or worker's compensation regarding their heel pain. Patients will also be excluded if they are pregnant. All participants will sign an informed consent prior to participation in the study.

Following baseline examination, patients will be randomly assigned to receive manual therapy, exercise and ultrasound alone or combined with electrical dry needling. Concealed allocation will be conducted using a computer-generated randomized table of numbers. Individual and sequentially numbered index cards with the random assignment will be prepared for each of the data collection sites. The index cards will be folded and placed in sealed opaque envelopes. Blinded to the baseline examination, the treating physical therapist will open the envelope and proceed with treatment according to the group assignment.

All participants in both groups will receive up to eight treatment sessions at a frequency of once

or twice per week over a 4-week period. Both groups will receive an impairment-based manual therapy approach directed to the lower limb, an exercise program including self-stretching of the plantar fascia and the Achilles tendon and strengthening exercises for the intrinsic muscles of the foot, and therapeutic ultrasound (3 MHz, 1.5 W/cm^2, 20% duty cycle for 5 minutes to the most tender region of the proximal portion of the plantar fascia). Impairment-based manual therapy will be directed primarily to the foot/ankle, but also to the hip and knee, including but not limited to passive anterior to posterior talocrural joint mobilizations in weight-bearing and non-weight-bearing positions and/or distraction thrust talocrural joint manipulation to improve ankle dorsiflexion, subtalar joint lateral glide mobilizations for eversion and inversion, and anterior and posterior first tarsometatarsal joint glide mobilizations for pronation and supination of the midfoot. All patients in both groups will be instructed to complete a home exercise program during the 4-week treatment period. The home exercise program will consist of the same strengthening and stretching exercises prescribed in the clinic, but without supervision. Patients will be told to complete the home exercise program 3 times daily on the days that they do not receive supervised physical therapy in the clinic. Patients will be asked to monitor their compliance with the home exercise program using a logbook.

The experimental group will receive conventional physical therapy, as described above, plus electrical dry needling for 20 minutes. Dry needling will include the 8-point standardized electrical dry needling protocol. The technique will be performed with the patient in prone or side lying. Sterilized disposable stainless steel monofilament needles will be used with three sizes: 0.18 mm x 15 mm, 0.25 mm x 30 mm, 0.30 mm x 40 mm. The plantar and medial surface of the foot will be cleaned with alcohol. The following 8 needles will be inserted: (1) superoposterior and slightly lateral insertion angle toward the proximal attachment of the plantar fascia at the medial tubercle of the calcaneus. “Periosteal pecking” at or near the proximal attachment of the plantar fascia at the medial tubercle of the calcaneus, will be performed for 30 seconds over the most painful tender point at the medial calcaneal tubercle; (2) medial to lateral perpendicular insertion within the distal abductor hallucis, immediately plantar and proximal to the head of the first metatarsal—common myofascial trigger point location and perineural for the medial plantar nerve; (3) medial to lateral perpendicular insertion within the abductor hallucis, immediately plantar and distal to the base of the first metatarsal—common myofascial trigger point location and perineural for the medial plantar nerve; (4) medial to lateral perpendicular insertion, immediately inferior to the sustentaculum tali (approximately two fingerbreadths inferior to the inferior apex of the medial malleolus)—near the bifurcation point of the tibial nerve and posterior tibial artery; (5) medial to lateral perpendicular insertion in the depression midway between the prominence of the medial malleolus and the Achilles tendon—perineural point for the tibial nerve at the ankle; (6) plantar to dorsal perpendicular insertion in the mid belly of flexor digitorum brevis and quadratus plantae muscles, two to three fingerbreadths distal to the anterior and plantar border of the calcaneus—common myofascial trigger point within the flexor digitorum brevis and perineural stimulation for the lateral plantar nerve; (7) plantar to dorsal perpendicular insertion within the distal plantar aponeurosis near its attachment at the metatarsophalangeal plates, within the depression on sole of the foot one third of the distance from the tip of the second toe to the posterior calcaneus; (8) medial to lateral perpendicular insertion within the abductor hallucis, within the depression immediately plantar to the navicular tuberosity—common myofascial trigger point and perineural for the medial plantar nerve.

In addition to the 8-point standardized protocol, therapists will also be permitted to insert needles at up to 4 additional locations in the foot and/or lower leg based on the presence of trigger points, or the report of sensitivity by the patient. The medial head of the gastrocnemius is recommended as one of the four optional needle insertion sites. Following insertion, needles will be manipulated bi-directionally to elicit a sensation of aching, tingling, deep pressure, heaviness or warmth, and then left in situ for 20 mins with electric stimulation (ES-160 electrostimulator ITO co.) using a low frequency (2 Hz), moderate pulse duration (250 microseconds), biphasic continuous waveform at an intensity described by the patient as "mild to moderate".

The primary outcome will be first-step pain during the morning as measured by the Numeric Pain Rating Scale (NPRS). Secondary outcomes will include resting mean foot pain (NPRS), pain during activity (NPRS), the Lower Extremity Functional Scale (LEFS), the Foot Functional Index (FFI), medication intake, and the Global Rating of Change (GROC) at baseline, 1 week, 4 weeks and 3 months. Medication intake will be measured as the number of times the patient has taken prescription or over-the-counter analgesic or anti-inflammatory medication in the past week for their heel pain, with five options: (1) not at all, (2) once a week, (3) once every couple of days, (4) once or twice a day, or (5) three or more times a day. Medication intake will be assessed at baseline and at 3 months. In addition, the Global Rating of Change (GROC) will be collected at 1 week, 4 weeks and 3 months following the initial treatment session.

In order to detect treatment differences of 2 points on the main outcome (i.e. the MCID for NPRS on first-step pain), and assuming a standard deviation of 3 points, a 2-tailed test, an alpha level of 0.05 and a desired power of 90%, the estimated desired sample size is calculated to be at least 49 subjects per group. A dropout percentage of 10% is expected, so 53 patients will be included in each group.

Statistical analyses will be performed using SPSS and conducted according to intention-to-treat analysis for patients in the group to which they are first allocated. Mean, standard deviations and/or 95% confidence intervals will be calculated for each variable. Baseline demographic and clinical variables will be compared between both groups using independent Student t-tests for continuous data and $\chi 2$ tests of independence for categorical data. The effects of treatment on first-step pain intensity, resting foot pain, pain during activity, physical function, and related-disability will each examined with a 2-by-4 mixed model analyses of covariance (ANCOVA) with treatment group (conventional physical therapy vs. conventional physical plus electrical dry needling) as the between-subjects factor, time (baseline, 1 week, 4 weeks and 3 months follow-up) as the within-subjects factor, and adjusted for baseline data for evaluating between-groups differences. Separate ANCOVAs will be performed for first-step pain intensity, mean heel pain at rest, pain during activity, the Lower Extremity Functional Scale, the FFI Total Score, the FFI Pain Subscale, the FFI Disability Subscale, and the FFI Activity Limitation Subscale as the dependent variable. For each ANCOVA, the main hypothesis of interest will be the 2-way interaction (group by time) with a Bonferroni-corrected alpha of 0.0125 (4 time points). We will use $\chi 2$ tests to compare self-perceived improvement with GROC and changes in medication intake. To enable comparison of between-group effect sizes, standardized mean score differences (SMDs) will be calculated by dividing mean score differences between groups by the pooled standard deviation. Numbers needed to treat and 95% confidence intervals will also be calculated at the 3-months follow-up period using each definition for a successful outcome.
